# Diversity changes of rhizosphere and endophytic bacteria in *Allium senescens* L. under drought stress and rewatering

**DOI:** 10.3389/fpls.2025.1571736

**Published:** 2025-05-07

**Authors:** Xue Song, Haotian Li, Chuhan Fu, Jiahong Li, Jihong Xiang, Xuetong Sun, Jielin Liu, Ligang Qin

**Affiliations:** ^1^ Department of Grassland Science, College of Animal Science and Technology, Northeast Agricultural University, Harbin, China; ^2^ Grassland Station of Heilongjiang, Harbin, China; ^3^ Institute of Forage and Grassland Science, Heilong Academy of Agricultural Science, Harbin, China

**Keywords:** *Allium senescens* L., drought stress, endophytic bacteria community, rhizosphere bacteria community, *Streptomyces*

## Abstract

**Introduction:**

Drought stress severely impacts plant productivity, particularly in non-cultivated species such as *Allium senescens*.L. However, the role of rhizosphere and endophytic bacterial communities in enhancing drought tolerance remains underexplored.

**Methods:**

We used 16S rRNA amplicon sequencing to investigate microbial communities in the rhizosphere, roots, bulbs, and leaves of *A. senescens* under simulated drought conditions using PEG-6000 (CK, 5%, 15%, 25%) and post-rehydration recovery. Alpha and beta diversity, phylogenetic relationships, and functional predictions were analyzed.

**Results:**

Drought stress reduced rhizosphere bacterial diversity by 42% but increased leaf diversity by 52%. The 15% PEG treatment marked a key threshold for community shifts. *Streptomyces* and *Ralstonia* were significantly enriched under drought, and functional predictions indicated their involvement in osmotic regulation and phytohormone synthesis. Post-rehydration partially restored microbial composition in aerial tissues but not in the rhizosphere.

**Discussion:**

These findings suggest that drought induces niche-specific microbial adaptations and that bacterial community structure plays a critical role in drought resilience. This study provides insights into plant-microbe interactions and offers a basis for developing microbial strategies to improve drought tolerance in *Allium* species.

## 1 Introduction

Drought has the potential to disrupt the normal growth and development of plants (Li et al., 2020; Kvgk and Ramarao, 2019). More specifically, drought affects plant germination, growth, physiology, leaf water content, photosynthesis, stomatal function, chlorophyll content, flowering and maturity, plant height, and biomass (Todorova et al., 2022; Onyemaobi et al., 2021; Javed et al., 2022; Wan et al., 2022; Pour-Aboughadareh et al., 2020). Drought can lead to oxidative damage and membrane peroxidation in plants by disrupting osmotic regulation and signal transduction processes, resulting in toxic effects and triggering a cascade of issues, including a reduction in plant yield (Duke et al., 2017; Kumar et al., 2020). Studies have shown that drought affects the growth and development of *Allium* plants. Drought stress led to a substantial decline in both the absolute and relative water content of *Allium schoenoprasum* L. leaves. Additionally, it elevates the osmotic pressure within the leaf sap and intensifies leaf transpiration while concurrently suppressing the production of reactive oxygen species (Egert and Tevini, 2002). Investigations into the aldehyde dehydrogenase (*ALDH*) superfamily of garlic (*Allium sativum* L.) and colleagues revealed that drought conditions lead to the down-regulation of the majority of *AsALDH* genes (Twaij et al., 2023). AsTCP17 protein, as a positive regulator, was involved in drought resistance of *A. senescens* (Fu et al., 2023).

Endophytes are defined as bacteria or fungi that inhabit the intercellular spaces or compartments of various tissues and organs in healthy plants, either at a specific stage or throughout their entire life cycle (Hallmann et al., 1997). These endophytes have co-evolved over an extended period with their host plants, establishing a stable and mutually advantageous symbiotic relationship (Hardoim et al., 2015). Bacterial endophytes commonly inhabit intercellular spaces in plants (Hardoim et al., 2015). Endophytes are known to colonize intercellular spaces within various plant components such as roots, leaves, stems, flowers, and seeds (Kandel et al., 2015; Mitter et al., 2017; Glassner et al., 2017). The extent of colonization can range from being confined to specific tissues to encompass the entire plant. However, the question remains whether endophytes must migrate to particular organs or tissues to optimally fulfill the roles of the identified endophytes (Hardoim et al., 2015).

The plant rhizosphere microbial community is the first microbial layer of plant defense, followed by endophytes, as has been widely reported (Dini-Andreote, 2020; Zhang and Mason, 2022). The presence of many beneficial bacteria in the rhizosphere and roots of plants confers various benefits to plants, including increased plant biomass, improved plant nutrient uptake and utilization, improved plant stress tolerance, and better adaptation to the environment (Cao et al., 2023; Papik et al., 2020). Endophytes facilitate plant growth by fixing nitrogen, producing plant hormones, acquiring nutrients, and enhancing tolerance to abiotic and biotic stresses (Kandel et al., 2017).

Drought substantially modifies the biomass, diversity, and structure of the endosphere and rhizosphere microbial communities, often leading to changes or disturbances in ecosystem processes and plant community dynamics (Seaton et al., 2022; Naylor and Coleman-Derr, 2018; Naylor et al., 2017). In cases in which drought conditions do not result in irreversible harm to crops, prompt regulatory intervention can promote normal growth and development (De Vries et al., 2020; Mega et al., 2019). It is believed that endophytic bacteria in host plants improve their capacity to adapt to biotic and abiotic stress conditions. Drought selectively recruits more drought-tolerant plants depending on the bacteria, which benefits the host (Liu et al., 2021; Ullah et al., 2019). Beneficial microbes can produce permeable material through biofilm formation and undergo morphological changes to enhance the drought resistance capacity, increase the availability of soil nutrients, promote organic matter decomposition, and secrete plant hormones to enhance plant drought resistance (Marasco et al., 2021; Santos-Medellín et al., 2021).

Currently, limited research has been conducted on the drought resistance mechanisms of the *Allium* species. *Allium senescens* L. (*A. senescens*), one member of the *Allium* genus, exhibits robust drought tolerance coupled with a high degree of adaptability and diverse practical applications (Munim et al., 2023). Specifically, the *A. senescens* is used for its ornamental value (Zhang et al., 2014), but more importantly, it has a genetic link to the cultivated onion *Allium cepa* and can be used as a source of trait genes in breeding strategies (Mallor et al., 2014). However, the underlying mechanism of drought tolerance in *A. senescens* remains elusive, potentially involving alterations in the composition of the rhizosphere and the endophytic bacterial community. The specific dynamics of rhizosphere and endophytic bacterial community diversity respond to drought stress in this plant species have not been fully elucidated. Therefore, we investigated the impact of drought stress on the rhizosphere and endophytic bacterial communities of *A. senescens* using microbiome sequencing techniques to elucidate the mechanisms by which the bacteria enhance the drought tolerance of plant species. Meanwhile, this study may not only contribute to the mechanism of bacteria enhancing plant drought tolerance and enriches the significance of endophytic bacteria resources in *Allium* plants, but also has potential application value for agricultural production practice. We hypothesized that 1) the diversity of the rhizosphere bacterial community was higher than that of the endophytic bacterial community, 2) the drought resistance of *A. senescens* is related to rhizosphere and endophytic bacteria, and 3) drought stress will affect the rhizosphere and endophytic bacterial community of *A. senescens*.

## 2 Material and methods

### 2.1 Plant sampling and drought stress

High-quality seeds of *A. senescens*, sourced from the Key Laboratory of Forage Breeding at Northeast Agricultural University, underwent a two-step sterilization protocol to ensure uniformity and minimize contamination: immersion in 75% ethanol (v/v) for 5 minutes followed by 10% sodium hypochlorite solution (v/v) for 10 minutes. After thorough rinsing with distilled water and air-drying, the seeds were sown in sterile Petri dishes (50 seeds per dish) lined with filter paper and cultivated under controlled conditions (25°C, constant hydration) until germination. Seedlings reaching 10 cm in height were transplanted into pots filled with soil (8-10 seedlings per pot), with a total of 25 pots arranged in a lighted culture chamber maintained at 22°C, 3200 K light intensity, and controlled humidity, with weekly watering to sustain optimal growth. To simulate drought stress, 20 cm-tall seedlings were subjected to a gradient of polyethylene glycol (PEG-6000) concentrations: a control group (CK) irrigated with distilled water, three drought stress groups (D2: 5%, D3: 15%, D4: 25% PEG-6000), and four corresponding rehydration groups (R1-R4) that resumed regular watering for one week after a two-week drought period.

The experimental nutrient soil was procured from a Harbin City, Heilongjiang Province flower market. It consisted of peat soil, leaf mold, and vermiculite mixed in a ratio of 2:1:1.

Each treatment was replicated five times, with 500 mL of PEG-6000 solution or distilled water applied every six days. Drought treatments lasted for two weeks, followed by the rehydration phase for applicable groups. Treatment details are summarized in **Table 1**:

**Table 1:** Drought stress and recovery treatments.

Name	Treatment
CK	0% PEG-6000 for two weeks.
D2	5% PEG-6000 for two weeks.
D3	15% PEG-6000 for two weeks.
D4	25% PEG-6000 for two weeks.
R1	CK group with one additional week of regular watering.
R2	D2 group followed by one week of regular watering.
R3	D3 group followed by one week of regular watering.
R4	D4 group followed by one week of regular watering.

After the treatments, samples of leaves, bulbs, roots, and rhizosphere soil were collected from each group. Each sample type was collected in triplicate to ensure biological replicability. Samples were immediately flash-frozen in liquid nitrogen and stored at -80°C for subsequent analyses.

### 2.2 Experimental procedure

#### 2.2.1 DNA extraction and sample quality control

Rhizosphere soil genomic DNA was extracted using a TianGen magnetic bead soil genomic DNA extraction kit (TianGen, BeiJing, China). Genomic DNA was isolated from roots, bulbs, and leaves using the Hexadecyl trimethyl ammonium bromide (CTAB) method. The purity and concentration of DNA were determined using 1% agarose gel electrophoresis. To achieve a suitable concentration for subsequent analyses, an aliquot of the DNA sample was placed in a centrifuge tube and diluted with sterile water to a final concentration of 1 ng/μL.

#### 2.2.2 Amplicon generation

The V57 region of the 16S rRNA gene was amplified by polymerase chain reaction (PCR). The primer sequences used were 5’-AACMGGATTAGATACCCKG-3’ and 5’-ACGTCATCCCCCCACCTTCC-3.’ Each PCR mixture was prepared by adding 15 μL of Phusion High-Fidelity PCR Master Mix (New England Biolabs, Ipswich, USA) along with 0.2 μM of each primer and a DNA template of 10 ng. The thermal cycling protocol began with a denaturation step at 98°C for 1 min, followed by 30 amplification cycles, including denaturation at 98°C for 10 s, annealing at 50°C for 30 s, and extension at 72°C for 30 s, and a final extension at 72°C for an additional 5 min.

#### 2.2.3 PCR products quantification and qualification

PCR products were analyzed using 2% agarose gel electrophoresis. Subsequently, the qualified products were purified with magnetic beads and their concentrations were determined by enzyme labeling. Equal amounts of these samples were combined based on PCR product concentrations. Thereafter, the mixed PCR products were analyzed by 2% agarose gel electrophoresis. To recover the target band, a universal DNA purification recovery kit (TianGen, BeiJing, China) was used.

#### 2.2.4 Library preparation and sequencing

Sequencing libraries were prepared using the NEB Next^®^ Ultra™ II FS DNA PCR-free Library Prep Kit (New England Biolabs, Ipswich, USA), following the manufacturer’s instructions, and incorporating index sequences. Library quality was assessed using a Qubit Fluorometer and validated via real-time PCR for quantification, as well as by electrophoresis on an Agilent Bioanalyzer (Novogene, BeiJing, China). Subsequently, the quantified libraries were pooled and sequenced on Illumina platforms (Novogene, BeiJing, China), with the procedure tailored to meet the desired library concentration and required data output volume.

#### 2.2.5 Amplicon sequence variants denoise and species annotation

Denoising was performed with DADA2 or the Deblur module in the Qiime 2 software to obtain initial Amplicon Sequence Variants (ASVs). Species annotation was performed using the Qiime 2 software. The Silva Database was used as the annotation database. Multiple sequence alignment was performed using the Qiime 2 software. The absolute abundance of ASVs was normalized using a standard sequence number corresponding to the sample with the lowest number of sequences. Subsequent analyses of alpha and beta diversity were performed based on the output-normalized data.

### 2.3 Statistical analysis

This study employed Python 3.6.13, R 4.0.3(Lucent, USA), and Perl 5.26.2 were used for data analysis and mapping throughout the analysis process. The distribution histogram of the relative abundance in Perl was drawn using the SVG function. A heatmap was generated in R using the pheatmap function. Flower diagrams were produced perl with SVG function. One hundred genera with the highest abundance in the samples were selected and performed sequence alignment to draw the phylogenetic tree in perl with SVG function. To analyze the alpha diversity, richness, and uniformity of the communities in the sample, alpha and beta diversity were calculated using Qiime 2. The UPGMA map was drawn using the upgma.tre function in Qiime 2, and a clustering tree was constructed on the UPGMA based on the weighted UniFrac distance matrix. Principal coordinates analysis (PCoA) and non-metric multidimensional scaling (NMDS) analyses were performed using the ade4 and ggplot2 packages in R 4.0.3. Linear discriminant analysis effect size (LEfSe) analysis was used an exclusive package called Lefse.

## 3 Results

### 3.1 Species composition of bacterial community in *A. senescens*

**Figure 1:**
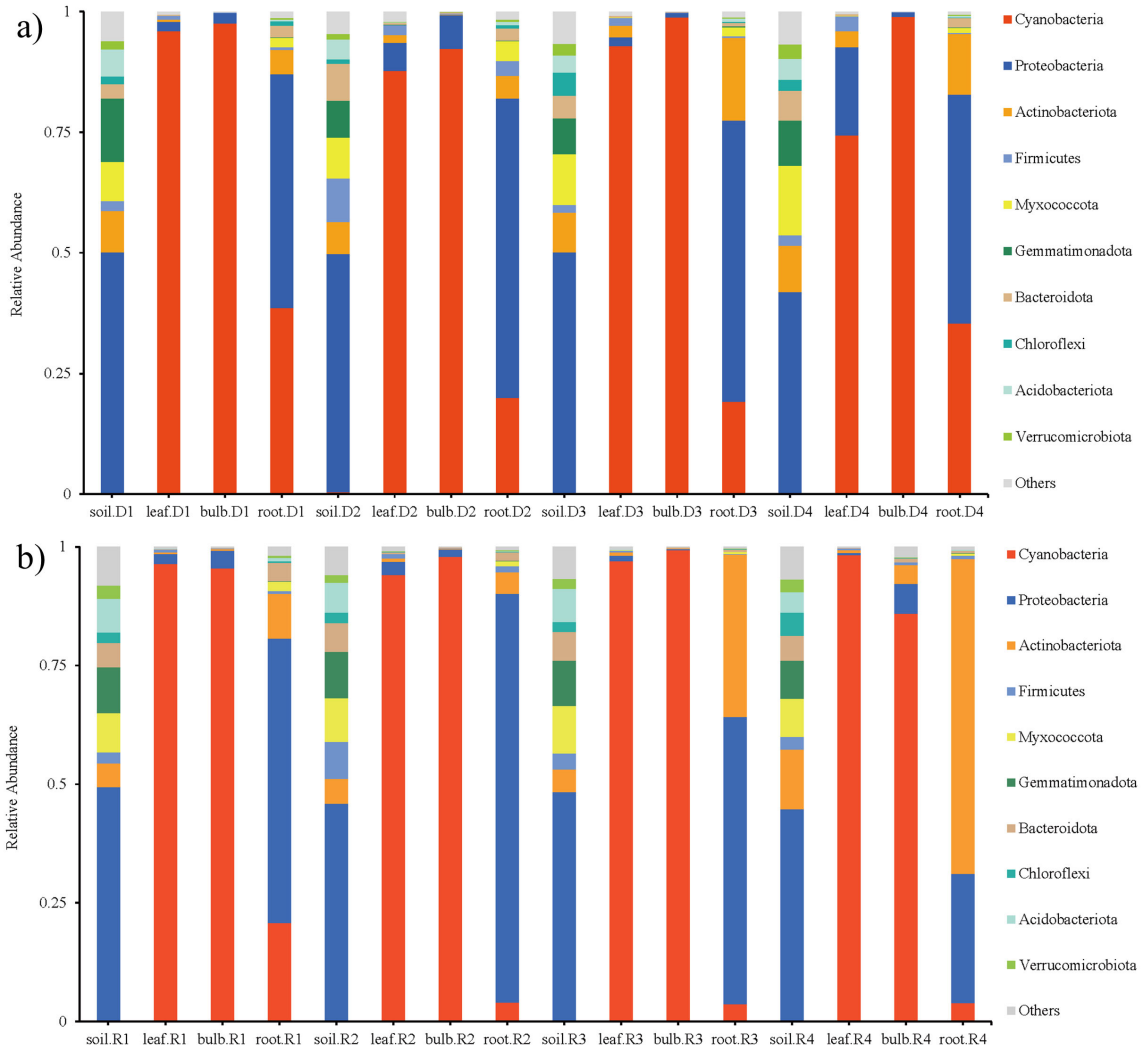
Species abundance of endophytic bacteria in *A*. *senescens* after drought stress **(a)** and re-watering **(b)** at the phylum level.

**Figure 2:**
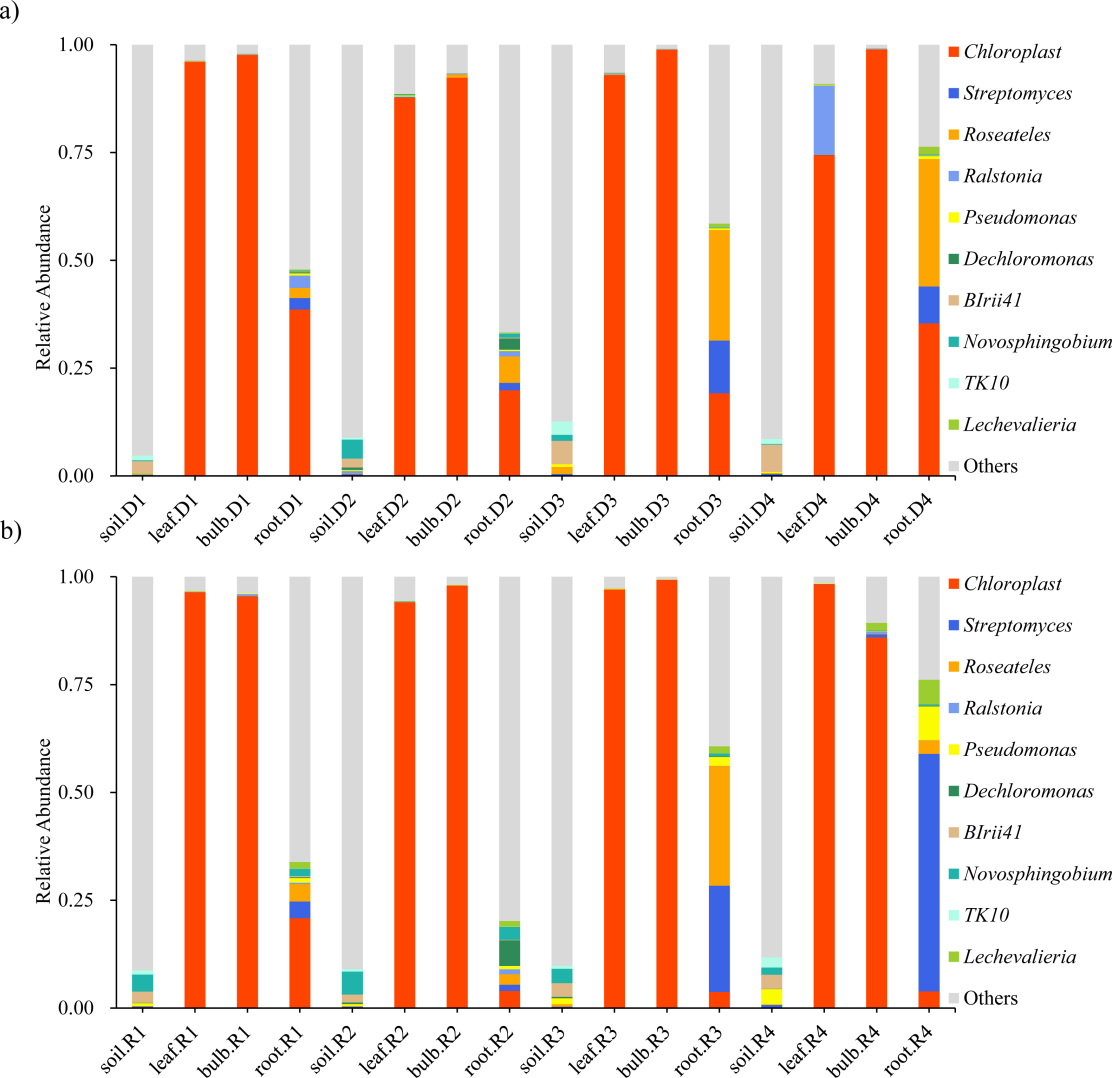
Species abundance of endophytic bacteria in *A*. *senescens* after drought stress **(a)** and re-watering **(b)** at the genus level.

High-throughput sequencing identified 45 phyla, 126 classes, and 944 genera across rhizosphere soil, roots, bulbs, and leaves of *A. senescens*. Cyanobacteriota were dominant in non-stressed leaves (98%) and bulbs (96%), whereas Pseudomonadota prevailed in roots (48%) and rhizosphere soil (50%) (**Figures 1a, 1b**). Drought stress induced significant taxonomic shifts: Cyanobacteriota abundance declined across all tissues, particularly in leaves (98% to 82% under 25% PEG), while Actinobacteria increased in leaves (CK: 0.5% vs. D3: 8.2%) and Bacillota in bulbs (CK: 1.1% vs. D3: 4.7%) (**Figure 1a**). At the genus level, *Chloroplast* (51.5%) and *Streptomyces* (3.6%) were dominant in control plants (**Figures 2a, 2b**). The term “*Chloroplast*” refers to sequences associated with photosynthetic bacteria, such as Cyanobacteriota, likely derived from *chloroplast* DNA detected in the samples. Under 15% PEG treatment, *Streptomyces* surged to 8.4% in roots (*P* < 0.001), while *Ralstonia* increased 2.3-fold in leaves (**Figure 2a**). The enrichment of *Streptomyces* suggests its potential role in drought adaptation through osmolyte production and phytohormone regulation.

### 3.2 Bacterial community structure and composition of *A. senescens*

**Figure 3:**
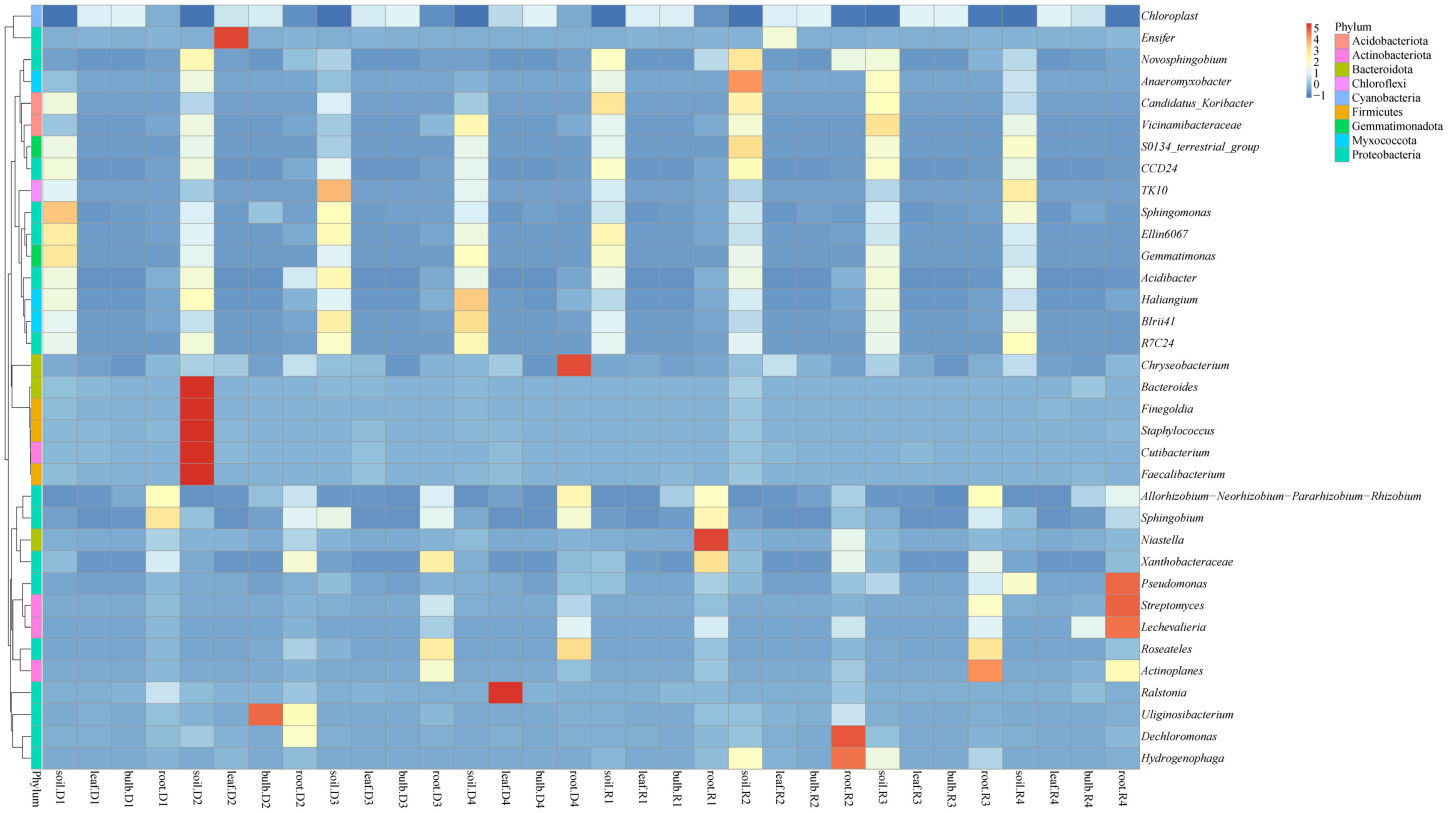
Cluster heat map of species abundance at the genus level of *A. senescens* after drought stress and re-watering.

Heatmap analysis highlighted distinct microbial niches, with *Gemmatimonas* and *Haliangium* uniquely enriched in the rhizosphere, potentially involved in soil nutrient cycling (**Figure 3**). Bulbs and leaves shared 68% of ASVs, including *Ensifer*, also known as *Sinorhizobium*, suggesting potential nitrogen-fixing roles in aerial tissues. Drought stress amplified niche-specific enrichment: *Ensifer* (leaves), *Uliginosibacterium* (bulbs), and *Bacteroides* (rhizosphere) were significantly enriched under 5% PEG (**Figure 3**). Post-rehydration, these taxa declined, but *Anaeromyxobacter* and *Pseudomonas* persisted in roots, indicating their potential involvement in stress resilience (**Figure 3**).

**Figure 4:**
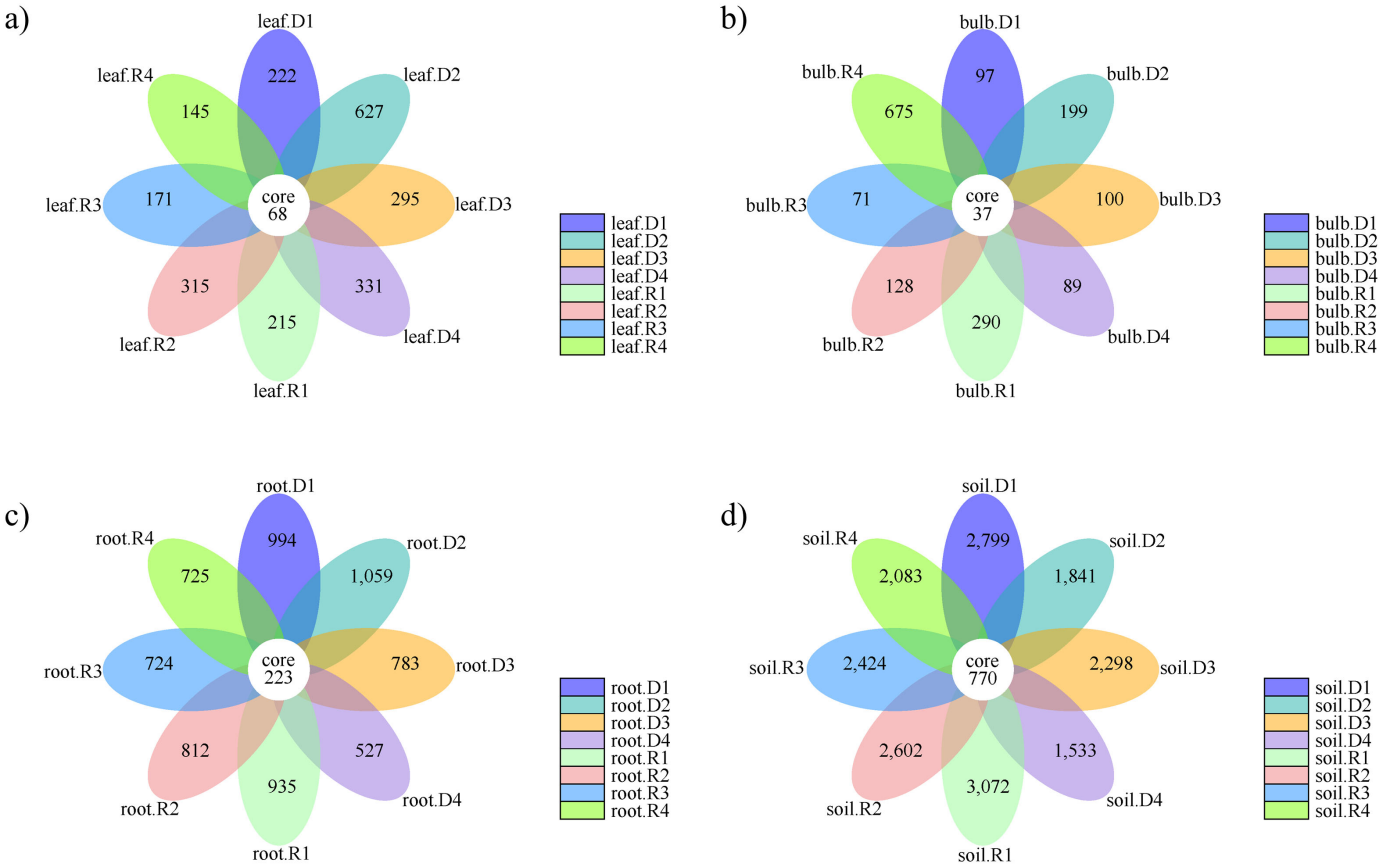
Number of ASVs in the bacterial community of *A*. *senescens* after drought stress and re-watering. **(a)** Flower plot of leaf of *A*. *senescens*, **(b)** Flower plot of bulb of *A*. *senescens*, **(c)** Flower plot of root of *A*. *senescens*, **(d)** Flower plot of rhizosphere soil of *A*. *senescens*.

The composition of the endophytic bacterial communities within different parts of *A. senescens* exhibited a higher degree of similarity (**Figure 4**). The leaves of *A. senescens* contained 68 ASVs, and drought stress induced the development of distinct ASVs, particularly in the 15% and 25% drought treatment groups, where rehydration correspondingly reduced the ASVs count (**Figure 4a**). The bulbs of *A. senescens* harbored 37 shared ASVs, with drought stress having a minimal impact on this community (**Figure 4b**). Application of drought stress at 5% and 15% resulted in increased bulb-associated ASVs, which conversely decreased after rewatering. At 25% drought stress, bulb ASVs decreased under drought conditions, but increased after rewatering. In the roots, 223 ASVs were identified as being shared (**Figure 4c**). Drought stress exceeding 15% led to a reduction in root-associated ASVs, which was temporarily alleviated by re-watering. The rhizosphere soil of *A. senescens* harbored 770 ASVs (**Figure 4d**). Drought stress significantly depleted the ASVs count in the rhizosphere soil, a condition that was not rectified following re-watering. Notably, the highest number of unique ASVs was identified in rhizosphere soil, followed by root, leaf, and bulb tissues.

**Figure 5:**
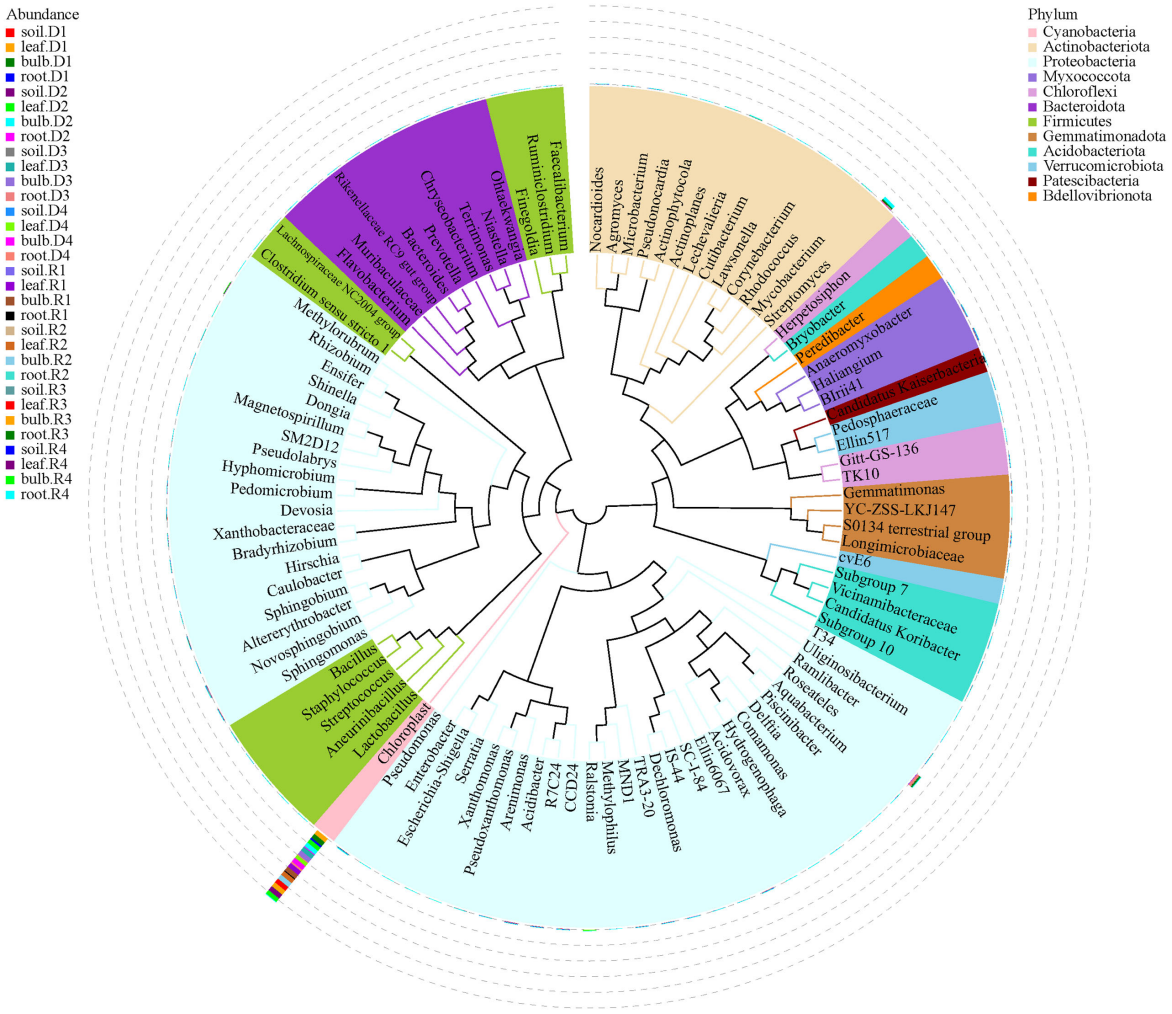
Phylogenetic tree of endophytic bacteria in *A. senescens* under drought stress and re-watering. The phylogenetic tree was constructed using the representative sequence of the genus-level species; the color of the branch and the fan represents its corresponding door, and the stacking histogram outside the fan ring represents the abundance distribution information of the genus in different samples. (The left legend is the sample information, and the right legend is the classification information at the phylum level corresponding to the genus level species).

To investigate the phylogenetic relationships among genus-level species, we retrieved representative sequences from the top 100 genera using multiple sequence alignment (**Figure 5**). The majority of the bacteria within *A. senescens* were classified as Pseudomonadota, with a considerable number of strains also belonging to Actinobacteria, Bacteroidetes, and Bacillota.

### 3.3 The α diversity of bacteria in *A. senescens*

Alpha diversity indices exhibited tissue- and treatment-specific trends (**Table 2**). Rhizosphere soil displayed the highest species richness (Chao1: 850 ± 32), which declined by 42% under 15% PEG (493 ± 28; *P* < 0.01). Conversely, bulb diversity transiently increased at 5% PEG (Chao1: 162 ± 12 vs. CK: 120 ± 15; *P* < 0.05) but dropped at 25% PEG (89 ± 10; *P* < 0.01). Leaves showed elevated Shannon diversity under drought (CK: 2.1 ± 0.2 vs. D3: 3.2 ± 0.3; *P* < 0.01), suggesting stress-induced microbial niche expansion. These trends indicate that microbial adaptation under drought varies across plant tissues, with rhizosphere soil experiencing the most pronounced diversity loss.

**Table 2:** The α diversity of bacteria in *A. senescens* after drought stress and rewatering.

Sample	Chao1	Dominance	Goods coverage	Observed features	Pielou e	Shannon	Simpson
soil.D1	7106.877	0.003	0.981	5152	0.794	9.792	0.997
leaf.D1	552.282	0.814	0.999	502	0.121	1.081	0.186
bulb.D1	257.524	0.886	1.000	235	0.080	0.628	0.114
root.D1	2510.523	0.151	0.994	2071*	0.496	5.466	0.849
soil.D2	5691.852	0.008	0.986	4061	0.760	9.113	0.992
leaf.D2	1161.641	0.663	0.998	1048	0.203	2.032	0.337
bulb.D2	401.620	0.781	0.999	380	0.140	1.202	0.219
root.D2	2779.367	0.074	0.994	2340**	0.580	6.497	0.926
soil.D3	5736.870***	0.003	0.986***	4666***	0.808	9.847	0.997
leaf.D3	672.200	0.719	0.999	640	0.177	1.645	0.281
bulb.D3	271.020	0.924	0.999	245	0.055	0.435	0.076
root.D3	1967.794	0.110***	0.996	1737	0.514***	5.528***	0.890***
soil.D4	4792.386***	0.004	0.990***	3972	0.805	9.623	0.996
leaf.D4	707.679	0.497**	0.999	660	0.240	2.247	0.503
bulb.D4	221.522	0.924	0.999	197	0.054	0.408	0.076
root.D4	1690.909	0.204	0.996	1348	0.397***	4.130***	0.796
soil.R1	7611.737	0.004	0.979	5494	0.784	9.743	0.996
leaf.R1	575.130	0.789	0.999	523	0.135	1.223	0.211
bulb.R1	485.154	0.783	0.999	453	0.132	1.161	0.217
root.R1	2190.560	0.060	0.996	2023*	0.605	6.643	0.940
soil.R2	6649.311	0.006	0.984	5184	0.786	9.699	0.994
leaf.R2	779.992	0.790	0.998	692	0.139	1.308	0.210
bulb.R2	298.020	0.903	0.999	275	0.071	0.573	0.097
root.R2	2294.735	0.176	0.995	1879	0.490	5.327	0.824
soil.R3	6584.813***	0.004	0.983***	4958	0.795	9.761	0.996
leaf.R3	490.039	0.825	0.999	454	0.120	1.055	0.175
bulb.R3	196.750	0.936	1.000	184	0.049	0.366	0.064
root.R3	1977.093	0.127***	0.995	1575	0.489***	5.193***	0.873***
soil.R4	5583.006***	0.003	0.987***	4472	0.813	9.860	0.997
leaf.R4	473.242	0.856	0.999	395	0.097	0.837	0.144
bulb.R4	900.305	0.588**	0.999	852	0.247	2.401	0.412***
root.R4	1818.446	0.212	0.996	1519	0.455***	4.809***	0.788

*: significant (*P* < 0.05), **: extremely significant (*P* < 0.01), ***: the most significant (*P* < 0.001).

### 3.4 The β diversity of endophytic bacteria in *A. senescens*

**Figure 6:**
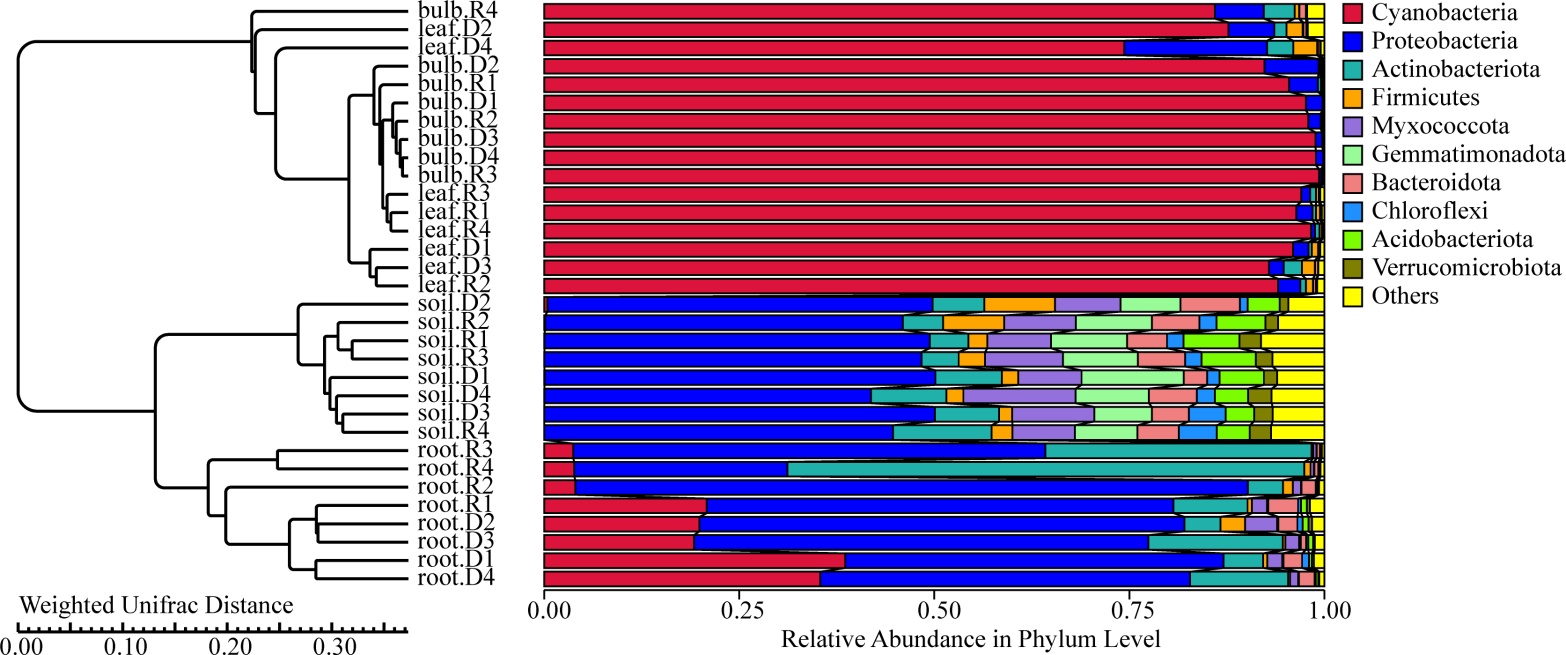
UPGMA dendrogram of *A. senescens* bacteria after drought stress and re-watering based on Weighted Unifrac distance.

**Figure 7:**
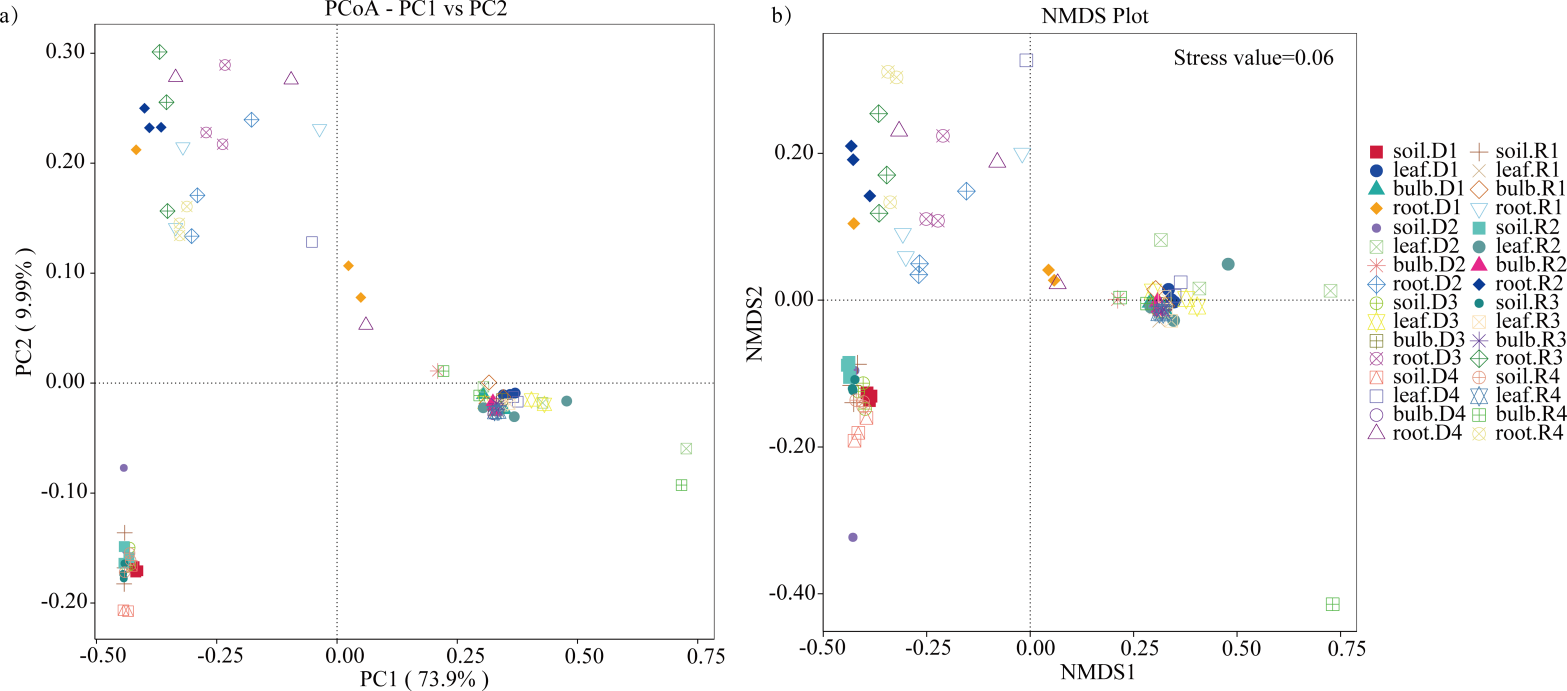
β-diversity of bacteria in *A*. *senescens* after drought stress and re-watering. **(a)** PCoA analysis of bacterial community in *A*. *senescens*, **(b)** NMDS analysis of bacterial community in *A*. *senescens*.

PCoA based on unweighted UniFrac distances revealed significant microbial differentiation across tissues (PERMANOVA, R² = 0.67, *P* = 0.001), with the first two axes explaining 83.9% of variance (**Figure 6a**). Drought stress amplified divergence, particularly in roots (**Figure 6b**). NMDS analysis (Stress value = 0.06) confirmed tighter clustering of bulb and leaf microbiomes, contrasting with rhizosphere heterogeneity (**Figure 6b**). UPGMA clustering further highlighted tissue-specific patterns, with roots and rhizosphere forming distinct clades (**Figure 7**). These findings suggest that drought disrupts microbial stability in belowground compartments while aerial tissues maintain greater compositional consistency.

### 3.5 Statistical test analysis of endophytic bacteria in *A. senescens*

**Figure 8:**
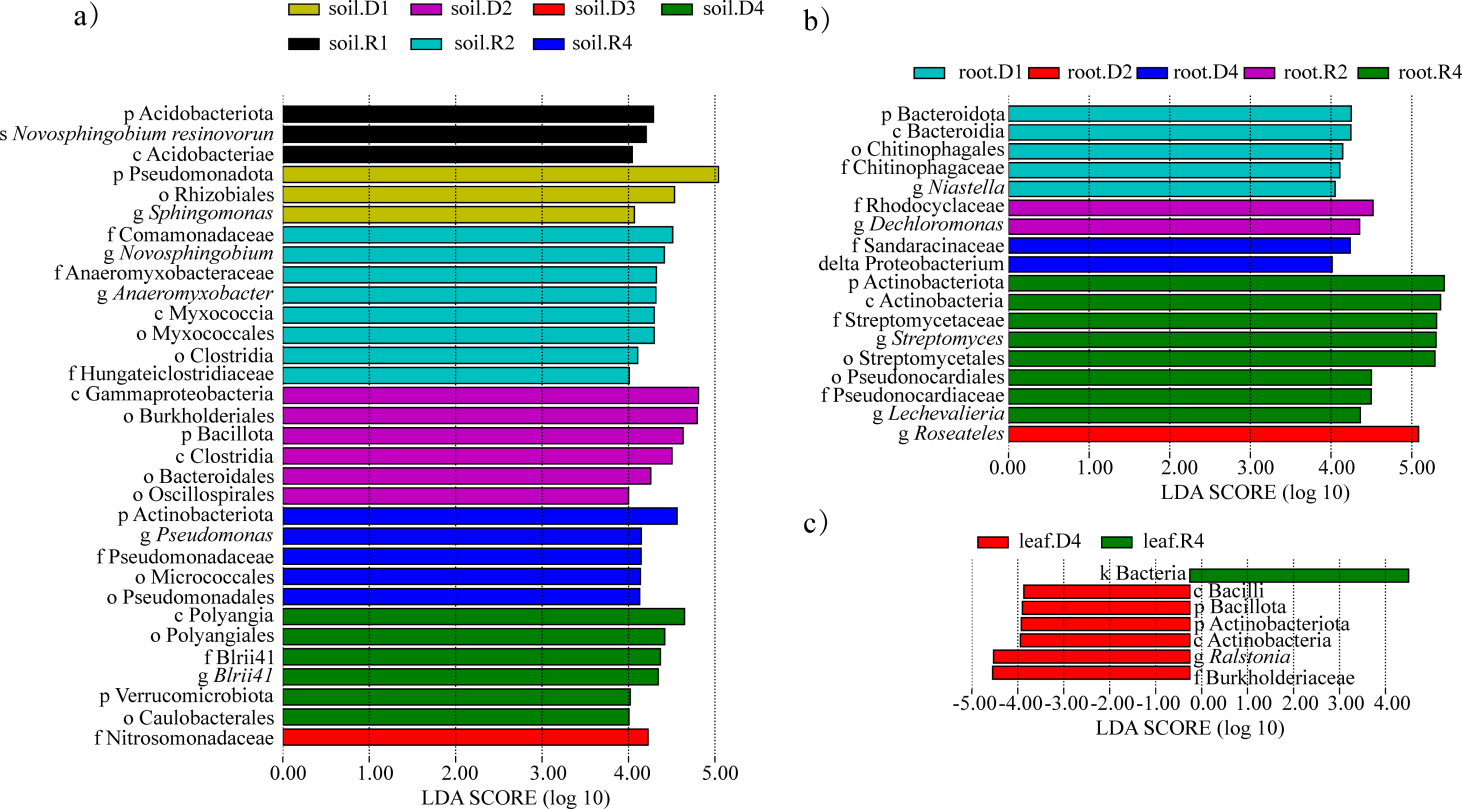
Significantly different bacteria in *A*. *senescens* after drought stress and re-watering. **(a)** LEfSe analysis of bacterial community in rhizosphere soil, **(b)** LEfSe analysis of bacterial community in root, **(c)** LEfSe analysis of bacterial community in leaf.

LEfSe identified 32 drought-responsive taxa (LDA > 4.0). In rhizosphere soil, Myxococcota (class: Deltaproteobacteria) and Bacillaceae were enriched under 15% PEG (**Figure 8a**). Roots exhibited Sandaracinaceae dominance at 5% PEG, while leaves under 25% PEG showed Bacilli and *Ralstonia* enrichment (**Figures 8b, c**). Post-rehydration, Actinoplanes and Hydrogenophaga became signature taxa in roots (**Figure 8b**). The persistence of Actinoplanes suggests its role in post-drought recovery, potentially through secondary metabolite production.

### 3.6 Bacterial network analysis of *A. senescens*

**Figure 9:**
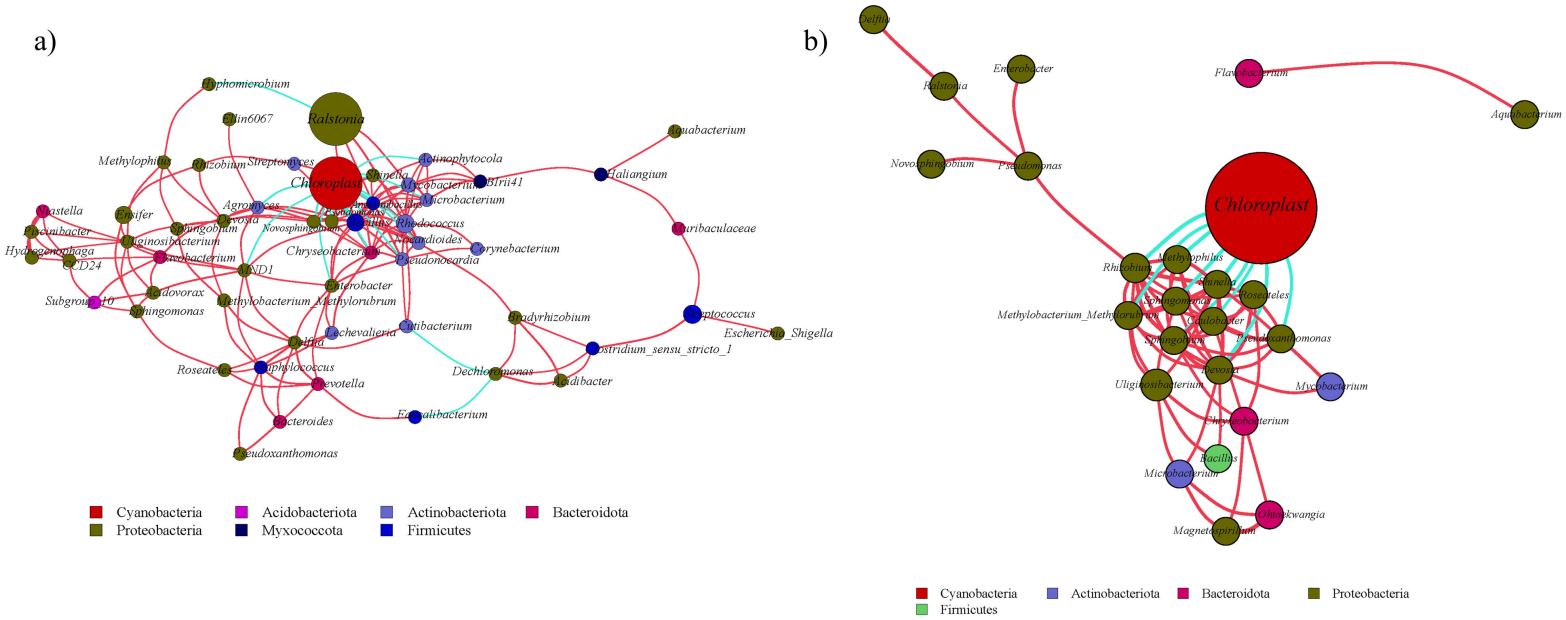
Analysis of endophytic bacterial network in the aboveground part of *A*. *senescens* after drought stress and re-watering. **(a)** Correlation network analysis of bacterial community in leaf of *A*. *senescens*, **(b)** Correlation network analysis of bacterial community in bulb of *A*. *senescens*.

**Figure 10:**
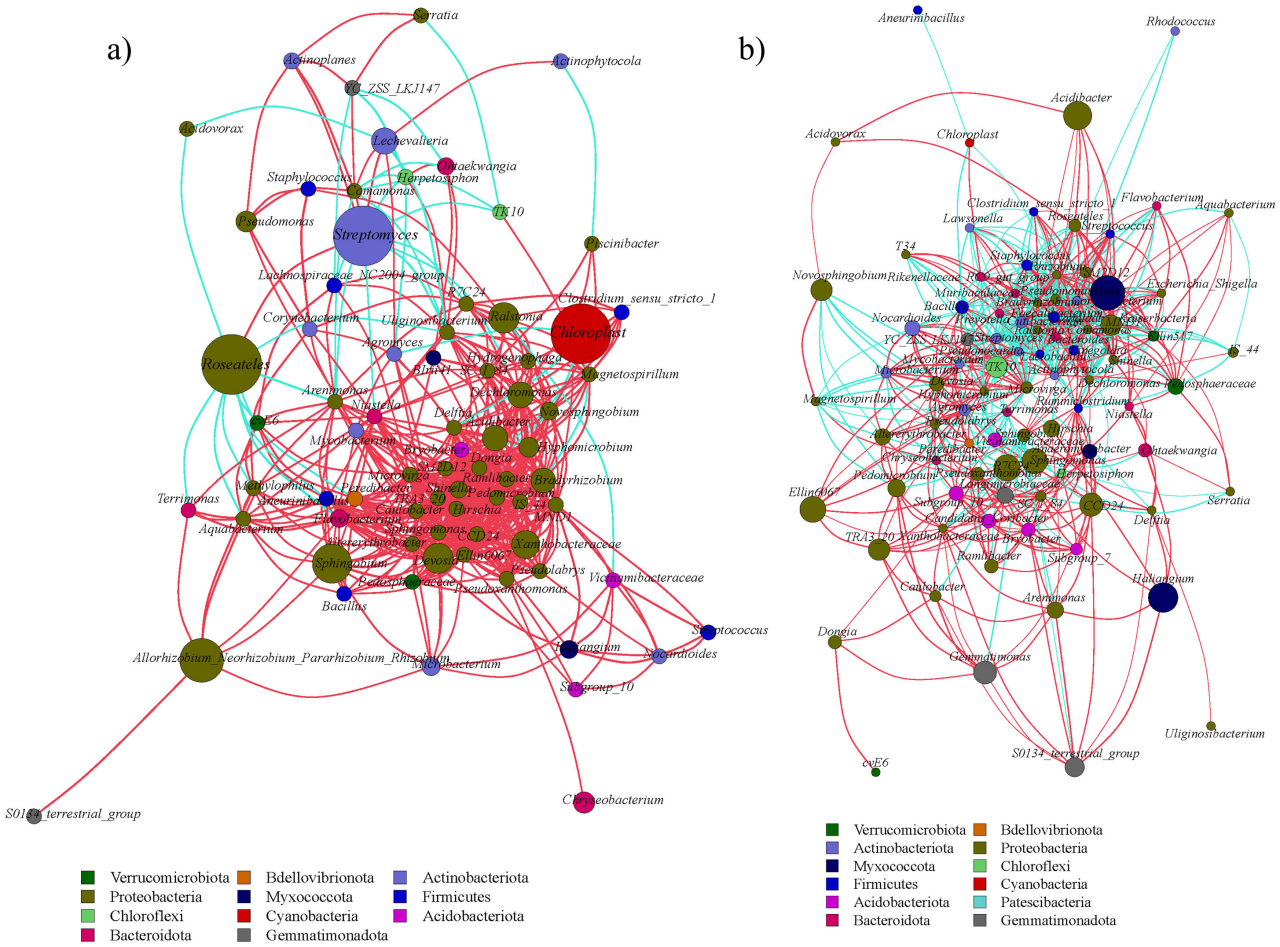
Analysis of bacterial network in the underground part of *A*. *senescens* after drought stress and re-watering. **(a)** Correlation network analysis of bacterial community in root of *A*. *senescens*, **(b)** Correlation network analysis of bacterial community in rhizosphere soil of *A*. *senescens*.

Network analysis revealed tissue-specific interaction patterns. In leaves, *Niastella* correlated positively with *Hydrogenophaga* (R = 1.0, *P* < 0.01), while Bacillus negatively linked to *Chloroplast* (R = -0.94, *P* < 0.05) (**Figure 9a**). In bulbs (**Figure 9b**), Cutibacterium and Hirschia showed a positive correlation (R = 1.0, *P* < 0.01), and Rhizobium and Chloroplast showed the maximum negative correlation (R = -0.87, *P* < 0.05). In roots, Prevotella and Methylorubrum showed the largest positive correlation (R = 1.0, *P* < 0.01), and Niastella and Roseateles showed the largest negative correlation (R = -0.87, *P* < 0.05) (**Figure 10a**). Rhizosphere soil displayed strong antagonism between *Finegoldia and BIrii41* (R = -0.96, *P* < 0.01) (**Figure 10b**).

### 3.7 Function prediction of endophytic bacteria in *A. senescens*

**Figure 11:**
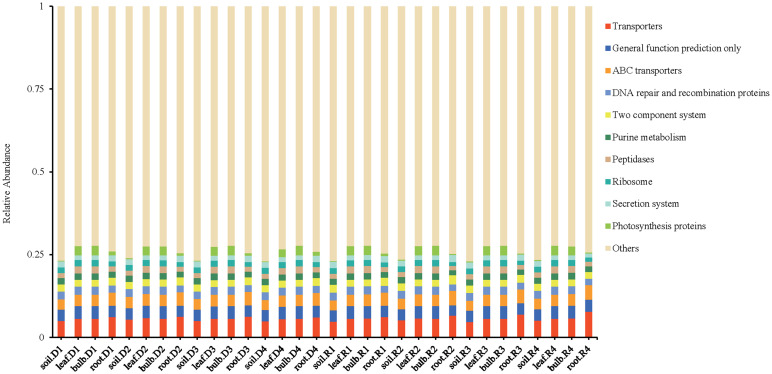
PICRUSt function prediction of bacteria in *A. senescens* after drought stress and re-watering.

PICRUSt functional predictions indicated significant metabolic shifts under drought. Pathways associated with transporters (5.71% of predicted functions), DNA repair (2.32%), and photosynthetic proteins (1.63%) were enriched, suggesting enhanced microbial adaptation mechanisms (**Figure 11**). Notably, *Streptomyces*-associated phenylalanine biosynthesis pathways were significantly upregulated under drought conditions, aligning with its role in stress adaptation.

## 4 Discussion

Our study provides a comprehensive analysis of how drought stress and subsequent rehydration reshape the rhizosphere and endophytic bacterial communities in *A. senescens*, a species renowned for its drought resilience. By employing high-throughput sequencing, we revealed tissue-specific microbial dynamics: drought reduced bacterial diversity in rhizosphere soil and roots but increased abundance in bulbs and leaves. These findings align with recent reports on *Allium sativum* (Munim et al., 2023), suggesting a conserved strategy among *Allium* species to reallocate microbial resources under stress. Notably, 15% PEG-6000 emerged as a critical threshold, inducing the most pronounced shifts in community structure (**Figure 4**). This parallels observations in crops like sugarcane (Liu et al., 2021), where moderate drought intensity triggers microbial functional remodeling. Compared with the non-drought stress group, under 15% -PEG stress treatment, the abundance of *Streptomyces*, *Roseateles*, *Pseudomonas* and *TK10* increased at the genus level, which was an important reason for the drought resistance of *A. senescens*. *Streptomyces* and *Pseudomonas* are generally accepted as drought-tolerant bacterial genera that form close associations with plants to provide host resilience to drought stress (Liu et al., 2024; Khan et al., 2025). It was found that *Roseateles* had greater resilience under osmotic stress (Lengrand et al., 2024). In addition, *TK10* also can resist abiotic stress (Wang et al., 2024). At the same time, another important reason for the drought resistance of *A. senescens* is the change of rhizosphere and endophytic bacterial community diversity. Alpha analysis (**Table 2**) showed that under 15% -PEG stress treatment, the Shannon index of rhizosphere soil, root and leaf bacterial communities of *A. senescens* became larger, and the stability of the community was enhanced, thus improving the ability of *A. senescens* to resist drought stress (Eisenhauer et al., 2012).

However, unlike *Allium tuberosum* (Huang, 2019), *A. senescens* bulbs and leaves were overwhelmingly dominated by Cyanobacteriota (> 90%), potentially leveraging their nitrogen-fixing capacity (Papik et al., 2020) to offset drought-induced nutrient limitations. Conversely, Pseudomonadota dominated the rhizosphere (50%), likely facilitating nutrient acquisition through metabolic versatility, as evidenced by enriched pathways such as ABC transporters and two-component systems (**Figure 11**). These results highlight both conserved and species-specific adaptations within the *Allium* genus, underscoring the need for tailored studies on plant-microbe interactions in undercharacterized species. The latest research shows that *AsTCP17* can upregulate the AtSVP expression, a key transcription factor gene in the ABA pathway, to enhance the drought-tolerance of the *A. senescens* (Fu et al., 2023). Further studies have found that CPMMV virus small interfering RNA (vsiRNA) enhances drought tolerance by targeting the host gene *PvTCP2*, reducing its transcriptional repressor, promoting autophagy and stomatal opening (Wu et al., 2024). The mechanism of TCP transcription factors family interacting with microorganisms to improve plant drought tolerance needs further study.

Central to our findings is the drought-induced enrichment of *Streptomyces*, a genus whose relative abundance surged 3.2-fold in rhizosphere soil and 2.5-fold in leaves under 15% PEG treatment (**Figures 8a, c**). This aligns with its documented role in enhancing drought tolerance in crops like sugarcane (Pang et al., 2024) and rice (Liu et al., 2024). Functional predictions (**Figure 11**) suggest *Streptomyces* may bolster host resilience through osmoprotectant synthesis (e.g., proline via *proB*), phytohormone modulation (e.g., IAA-driven root elongation), and pathogen suppression via antibiotic production. Equally notable was the incomplete recovery of certain taxa post-rehydration, such as rhizosphere *BIrii41* and leaf *Roseateles* (**Figure 4**). This hysteresis effect, previously observed in maize (Santos-Medellín et al., 2021), implies that drought may impose lasting impacts on microbial network stability, potentially affecting plant recovery trajectories. Such insights challenge the assumption of full microbial resilience after stress alleviation and emphasize the need to integrate temporal dynamics into drought adaptation models.

While our study advances understanding of *A. senescens*-microbe interactions, several limitations merit consideration. First, focusing solely on bacterial communities overlooks the potential contributions of fungi, particularly arbuscular mycorrhizal fungi known to enhance drought tolerance (Dini-Andreote, 2020). Future work should adopt a multi-kingdom approach, combining ITS and 16S sequencing. Second, although PICRUSt predictions offer functional insights, empirical validation through strain isolation and in *planta* assays is critical. For instance, targeted cultivation of *Streptomyces* sp. AS15 could clarify its role in phenylalanine biosynthesis—a pathway linked to drought resistance (Pang et al., 2024). Finally, controlled greenhouse conditions may oversimplify field realities; validating these findings in natural arid ecosystems, where soil heterogeneity and microbial competition prevail, remains essential.

Translating these discoveries into practice holds promise for sustainable agriculture. The drought-responsive taxa identified here, particularly *Streptomyces* and *Roseateles*, represent prime candidates for bioinoculant development. Field trials could assess their efficacy in enhancing yield stability in *Allium* crops under water-limited conditions. Moreover, the partial post-drought recovery of rhizosphere communities (**Figure 4d**) suggests that microbial management—such as targeted amendments during early recovery phases—could accelerate ecosystem rehabilitation. Integrating these microbial strategies with breeding programs, perhaps via CRISPR editing to optimize plant-microbe synergy, may further bolster drought resilience.

In conclusion, our work elucidates the dynamic interplay between *A. senescens* and its microbiome under drought stress, revealing both universal and novel adaptation mechanisms. By bridging ecological observations with functional predictions, we provide a framework for harnessing microbial resources to mitigate climate-driven agricultural challenges. Future studies integrating multi-omics approaches and field validation will be pivotal in unlocking the full potential of plant-microbe partnerships in arid agroecosystems.

## 5 Conclusion

In this study, we investigated the effects of drought stress and re-watering on the diversity and composition of bacterial communities in the leaves, bulbs, roots, and rhizosphere soil of *A. senescens*. The dominant bacteria in each niche were different, and the diversity of rhizosphere bacteria was much higher than that of the endophytic bacteria. The composition and diversity of the bacterial rhizosphere and endophytic bacteria were affected by drought and re-watering, and 15% PEG-6000 treatment had the greatest effect on the diversity of the bacterial rhizosphere and endophytic bacteria. *Roseateles*, *Streptomyces*, and other bacteria are important regulators of drought resistance in *A. senescens*. This study enhances our understanding of the rhizosphere and endophytic bacterial communities in *A. senescens*.

## Data Availability

The datasets generated for this study can be found in the NCBI Sequence Reads Archive (SRA) with the accession numbers SAMN47255586-SAMN47255681 for 16S rRNA, under the BioProject, PRJNA1230695.
